# Non-western immigrants' satisfaction with the general practitioners' services in Oslo, Norway

**DOI:** 10.1186/1475-9276-7-7

**Published:** 2008-02-27

**Authors:** Else Lien, Per Nafstad, Elin O Rosvold

**Affiliations:** 1 Norwegian Centre for Minority Health Research, Ullevaal University Hospital, Oslo, Norway; 2 Institute of General Practice and Community Medicine, Faculty of Medicine, University of Oslo, Norway

## Abstract

**Background:**

Over the last few years the number of immigrants from the non-western parts of the world living in Oslo, has increased considerably. We need to know if these immigrants are satisfied with the health services they are offered. The aim of this study was to assess whether the immigrants' level of satisfaction with visits to general practitioners was comparable with that for ethnic Norwegians.

**Methods:**

Two population-based surveys, the Oslo Health Study and the Oslo Immigrant Health Study, were performed on selected groups of Oslo citizens in 2000 and 2002. The response rates were 46% and 33%, respectively. In all, 11936 Norwegians and 1102 non-western immigrants from the Oslo Health Study, and 1774 people from the Oslo Immigrant Health Study, were included in this analysis. Non-western immigrants' and ethnic Norwegians' level of satisfaction with visits to general practitioners were analysed with respect to age, gender, health, working status, and use of translators. Bivariate (Chi square) and multivariate analyses (logistic regression) were performed.

**Results:**

Most participants were either moderately or very satisfied with their last visit to a general practitioner. Non-western immigrants were less satisfied than Norwegians. Dissatisfaction among the immigrants was associated with young age, a feeling of not having good health, and coming from Turkey, Iran, Pakistan, or Vietnam as compared to Sri Lanka. The attendance rates in the surveys were rather low and lowest among the non-western immigrants.

**Conclusion:**

Although the degree of satisfaction with the primary health care was relatively high among the participants in these surveys, the non-western immigrants in this study were less satisfied than ethnic Norwegians with their last visit to a general practitioner. The rather low response rates opens for the possibility that the degree of satisfaction may not be representative for all immigrants.

## Background

There is a growing number of immigrants from non-western parts of the world in Oslo, the capital of Norway. The number increased from 10.1% to 16.9% of the total population, between 1993 and 2003, and by 01.01.2003 there were 87.472 immigrants living in Oslo [1]. These figures included people born in non-western countries as well as people born in Norway to parents born in non-western countries. The primary health care services in Oslo are affected by this growing number of immigrants.

The health problems of immigrants are influenced by culture and beliefs. Additionally, the immigrants' country of origin often do not have the same system of primary health care services as European countries [2]. The health of immigrants is also affected by genetic and biological factors, as well as language barriers, social, educational, and economic status [3,4]. Ethnocultural affiliation is also of importance [5].

Primary health care services are meant to be the foundation of the Norwegian health services [6]. Approximately 90% of the enquiries to general practitioners (GPs) are treated by the primary health care services. The immigrants' different backgrounds may lead to expectations to the primary health care services that personnel not yet have learnt to deal with. In addition, the immigrants' health needs may be different from those of ethnic Norwegians.

Two former studies in Oslo have shown that non-western immigrants usually thought they had received the health services they needed [7,8]. However, differences in requirements might be due to the need of a translator, the GP's understanding of the importance of patients' cultures and religions, as well as understanding the health problems that result from migration and refugee status. It is also of importance that the health workers have a knowledge of the different genetic diseases that immigrants may have.

In the present paper we want to compare non-western immigrants' degree of satisfaction with GP services with that of ethnic Norwegians, and how this satisfaction is affected by gender, age, own description of health, and income-producing work.

## Methods

Two studies in Oslo are examined in this paper, The Oslo Health Study and The Oslo Immigrant Health Study. The Oslo Health Study, a population based cross sectional study, was carried out between May 2000 and September 2001 as a collaboration between the Norwegian Institute of Public Health, the University of Oslo, and the Oslo City Council [9]. Altogether 40888 persons residing in Oslo December 31, 1999 were invited to participate. The participants were from selected birth-cohorts (1924/25, 1940/41, 1954/55, 1960, and 1969/70). Detailed information about the participants is found elsewhere [9].

The participants in the Oslo Health Study were divided in groups of Norwegians (born in Norway), western immigrants (born in western countries), and non-western immigrants (born in non-western countries). This categorization was done because of the small numbers of immigrant participants [10]. The participants from the non-western parts of the world originated from the following regions: Eastern Europe, North – Africa, Subsaharian Africa, Middle-East, Indian subcontinent, East- Asia, the Pacific, Middle- and South – America [10]. In this article the answers from these participants are compared to those from the Norwegian participants.

In 2002 the Oslo Immigrant Health Study was carried out as collaboration between the Norwegian Institute of Public Health and the University of Oslo [11]. Inhabitants born in Iran, Vietnam, Sri Lanka, and Turkey were invited to participate. In addition a 30% random sample of the Pakistanis was invited. The invited persons were born between 1942 and 1982 [11]. In total 11946 immigrants were eligible for invitation, and 11389 were reached by mail. The nine birth cohorts which were included in the Oslo Health Study, were not included in the immigrant study.

The two studies followed the same protocol including a physical screening (measures of blood pressure, weight, height etc), blood samples, and questionnaires. The Oslo Health Study included a main questionnaire, a first and a second supplementary questionnaire. The immigrant study included a main and a supplementary questionnaire. This article focuses on the answers to the questions in the first supplementary questionnaires in both surveys. The questionnaires can be found with official English translation [12].

In all 18770 persons (46%) participated in the Oslo Health Study. Totally 15282 persons answered the first supplementary questionnaire [9]. All the Norwegians (n = 11936) and the non-western immigrants (n = 1102) were included in our study. In the Oslo Immigrant Health Study 3726 persons (33%) participated, and 1774 persons answered the first supplementary questionnaire [11]. The attendance rates in the surveys were rather low and lowest among the non-western immigrants.

The main question we look into in this article is "How satisfied were you with the last visit to a doctor? (excluding hospital/outpatient department)". Three answers were possible : Very satisfied, moderately satisfied, and not satisfied. We look into the degree of satisfaction with the last visit to a general practitioner in relation to country of origin, gender, age, own description of health, need of translator, and income-producing work.

In order to examine the degree of satisfaction with the last visit to a general practitioner, we used the number of responders who had been to a general practitioner answering the question "What kind of doctor (excluding hospital and outpatient dept.) did you visit on the last occasion?" We also explored how many different general practitioners the responders had been treated by in the course of the last 12 months.

Age was divided into "old" (born before 1955) and "young" (born 1955 or later). The state of health was explored by the question "How would you describe your present state of health?" as this independent variable is an indicator of describing the level of health. Possible answers were: "Poor", "Not very good", "Good", and "Very good".

We also explored the need for translator "Does anyone help by translating for you when you visit the doctor?" giving five possible answers : "Have not been to a doctor in Norway", "Yes usually", "Sometimes", "No", and "Not relevant (do not need/want such help)".

The question "Are you currently employed ?" with the answers : "Yes, full time", "Yes, part time, and "No" was utilized in examining the degree of income-producing work. Persons who are unemployed or outside the labour force are at risk to develop psychological problems, and are therefore in need of health care services [13,14].

Two additional questions regarding use of health services were also explored: "Do you think that in Norway you have received the medical services you need?" with possible answers "Yes, always", "Yes and no", "No", "Have not needed such help", and "Don't know", and "Do you think the medical service you have received is better or not as good as a Norwegian would have received?" with the answers "Not as good treatment", "Same treatment", "Better treatment", "Have had no need", and "Don't know".

Bivariate (Chi square) and multivariate analyses (logistic regression) were performed by using SPSS 13.0. The level of significance was set to p ≤ 0.05. The studies are approved by the Norwegian Data Inspectorate and have been cleared by the Regional Committee for Medical Research Ethics.

## Results

The Oslo Health Study showed that 9949 people had been to a general practitioner (GP) on the last visit to a doctor. Of the 9829 people who answered the question about the level of satisfaction, 92.3% were ethnic Norwegians and 7.7% were non-western immigrants. In the Oslo Immigrant Health Study 1177 people had been to a GP on their last visit to a doctor, and 1157 people had answered the question about the level of satisfaction.

During the last year 82.2% of the ethnic Norwegians, and 82.1% of the non-western immigrants in the Oslo Health Study had visited one or more GPs. Of the Norwegians 46.1% and of the non-western immigrants 38.9% had seen only one GP. In the Oslo Immigrant Health Study 85.5% had been to one or more GPs during the last year, and 39.1% had seen only one GP.

The participants' degree of satisfaction with the last visit to a GP according to country of origin is shown in Table 1. The results showed that the Norwegians were predominantly very satisfied, while all the non-western immigrants were predominantly moderately satisfied in the studies.

**Table 1:** Degree of satisfaction (% of participants) with last visit to a general practitioner according to country of origin.

	The Oslo Health Study		The Oslo Immigrant Health Study					
	Norway	Non-western countries	Turkey	Sri Lanka	Iran	Pakistan	Vietnam	Total

	N = 9071	N = 758	N = 185	N = 312	N = 252	N = 186	N = 222	N = 1157

Very Satisf.	62.8	40.6	32.4	44.2	33.3	36.0	26.6	35,3
Mod. Satisf.	33.1	48.4	50.8	49.7	50.4	50.0	62.6	52,5
Not Satisf.	4.1	10.9	16.8	6.1	16.3	14.0	10.8	12,2

Analysis of satisfaction with respect to gender, age, own health, income-producing work, and use of translators are shown in Table 2. In relation to gender and income-producing work the Norwegians were mainly very satisfied while the non-western immigrants in both studies were mainly moderately satisfied. The results from the Oslo Health Study showed that more older than younger of the Norwegians, and of the non-western immigrants were very satisfied. In the Oslo Immigrant Health Study there were no age difference in being very satisfied, but the younger participants were more likely to be not satisfied with the services they had received.

**Table 2:** The degree of satisfaction (% of participants) with the last visit to a general practitioner

	The Oslo Health Study									The Oslo Immigrant Health Study				
	Norway				Non-western countries									
	Satisfaction				Satisfaction						Satisfaction			
	Very	Mod.	Not	N	Very	Mod.	Not	N	P*	Very	Mod.	Not	N	P*
Gender														
Women	64.9	31.5	3.6	5057	45.1	46.2	8.6	370	<0.001	34.4	53.1	12.5	561	Not sign.
Men	60.2	35.2	4.6	4014	36.3	50.5	13.1	388	<0.001	36.1	52.0	11.9	596	
														
Age**														
Young	55.3	39.5	5.2	5242	39.4	49.4	11.1	619	<0.001	35.4	51.2	13.3	914	0.04
Old	73.1	24.4	2.5	3829	46.0	43.9	10.1	139	<0.001	34.6	57.6	7.8	243	
														
Own health***														
Not good	59.7	34.8	5.5	1964	29.9	55.9	14.1	311	<0.001	27.3	55.0	17.8	484	<0.001
Acceptable	63.8	32.6	3.6	7003	47.9	43.3	8.7	436	<0.001	41.6	50.4	8.1	645	
														
Income-producing work														
Full time	58.0	37.4	4.6	5495	42.8	46.0	11.3	435	<0.001	37.9	51.6	10.5	607	0.02
Part time	64.8	32.7	2.5	921	35.4	54.9	9.8	82	<0.001	33.5	56.7	9.8	154	
Not working	62.0	32.2	5.7	950	33.9	54.2	11.9	177	<0.001	31.8	51.5	16.7	359	
														
Translation														
Yes					34.3	56.3	9.4	128		30.6	56.6	12.8	258	Not sign.
No					37.2	51.6	11.2	250		35.6	53.1	11.3	399	
Not relevant****					37.5	50.2	12.3	269		38.0	49.1	12.9	466	

* Significance testing (Chi Square): The Oslo Health Study: Comparison between Norwegians and non-western participants. The Oslo Immigrant Health Study: Comparison between the different categories.

** Old = born before 1955, Young = born in 1955 or later.

*** Own health: In the table the answers "poor" and "not very good" are included in the category "not good", and the answers "good" and "very good" are included in the category "acceptable".

**** Translation: The categories "yes usually" and "sometimes" are included in the category "yes". The category "have not been to a doctor in Norway" is excluded.

Most of the Norwegians were very satisfied, and this was independent of their state of health. The result from the Oslo Health Study showed that most of the non-western immigrants were very satisfied when their health was acceptable and only moderately satisfied when their health was not good. Most of the participants in the Oslo Immigrant Health Study were moderately satisfied, and this effect was independent of their state of health. Analysis by country showed that the when the state of health was "not good", 41.3% of the participants from Turkey were very satisfied, while only 22.2% of the participants from Vietnam were very satisfied.

The results of the logistic regression analysis confirmed the bivariate analysis of the Oslo Health Study (Table 3). It showed that dissatisfaction with the health services was

**Table 3:** Logistic regression analyses: Patients' satisfaction with their last GP-visit. Dependent variable: "very/moderately satisfied" (reference) versus "not satisfied".

	The Oslo Health Study			The Oslo Immigrant Health Study		
	N	OR(adjusted)	95% CI	N	OR(adjusted)	95% CI
Country I						
Norway	7306	ref				
Non-western	689	2.0	(1.5–2.7)			
						
Gender						
Women	4383	ref		534	ref	
Men	3612	1.4	(1.1–1.7)	570	1.1	(0.7–1.5)
						
Age						
Young	5767	2.9	(1.7–2.9)	874	2.5	(1.5–4.4)
Old	2228	ref		230	ref	
						
Own health						
Not good	1611	1.8	(1.4–2.3)	470	2.3	(1.6–3.5)
Acceptable	6384	ref		634	ref	
						
Income producing work						
Full time	5886	1.6	(1.1–2.4)	599	0.8	(0.5–1.2)
Part time	995	ref		160	0.6	(0.3–1.1)
Not working	1114	1.9	(1.3–3.0)	345	ref	
						
Country II						
Sri Lanka				299	ref	
Turkey				177	2.6	(1.4–5.0)
Iran				245	2.8	(1.5–5.1)
Pakistan				174	2.6	(1.4–5.0)
Vietnam				209	2.0	(1.1–3.9)

associated with being non-western, male, young, not in good health, and full time employed or unemployed rather than being part time employed.

Results from the Oslo Immigrant Health Study showed that dissatisfaction was associated with being younger, not in good health, and being from Turkey, Iran, Pakistan, or Vietnam as compared to Sri Lanka.

The participants in all immigrant groups were moderately satisfied independent of whether they needed a translator or not. Only 13 of the participants in the Oslo Health Study used a professional translator, and 104 of the participants used some other type of translator. Half of the first group were very satisfied with their last GP visit, and 1/3 of the other group were very satisfied. In the Oslo Immigrant Health Study 38 of the participants had used a professional interpreter, and 190 of the participants had used another translator. In the first group 2/5 were very satisfied and in the other group 1/3 were very satisfied with their last GP visit.

Of the non-western immigrants in the Oslo Health Study, 63.9% thought they had received the same health services as the Norwegians; 26% did not know. In the Oslo Immigrant Health Study 53.7% thought that they had received the same health services as the Norwegians, and 31.3% answered "don't know".

In the Oslo Health Study 42.4% of the non-western immigrants thought they had received the medical services they needed, and in the Oslo Immigrant Health Study 43.1% answered the same.

## Discussion

This study focuses on the degree of satisfaction with the visits to general practitioners in Oslo. A substantial proportion of participants were either very satisfied or moderately satisfied with their last visit to a GP. The degree of satisfaction was higher among the ethnic Norwegians than among the non-western immigrants even when factors such as gender, age, own health, and income-producing work were controlled for.

The attendance rates for these surveys were rather low, and lowest for the non-western immigrants. The first part of the questionnaires in both surveys contained some rather complex questions concerning eating habits which may have been difficult to answer [12]. This may have had a negative effect on the percentage of responders to the questions posed in this study.

Low participation rate is a major problem and challenge for most immigrant health studies [15]. Our studies are no exception, and this makes it difficult to draw firm conclusions. The results from the Oslo Health Study showed that self-selection according to socio-demographic variables had little impact on prevalence estimates [15]. In the Oslo Immigrant Health Study the nonresponder pattern was similar to that observed in the Oslo Health Study [16].

Low attendance rate among non-western immigrants can be due to language problems, cultural differences, and a lesser degree of integration. It seems reasonable to speculate that well integrated immigrants are more likely to participate in health surveys, and that the degree of satisfaction with health services could be dependent on degree of integration. This might imply that the degree of dissatisfaction is underestimated in our study. We found that the participants who described their health as not good were also the most dissatisfied. It might be that people with chronic illnesses, bad health in general, and those who receive disability benefit were less likely to answer the questionnaires as their lives are difficult, and they are less resourceful. This suggests that the percent of persons that were not satisfied with the general practitioner could have been higher, if the response rate had been better.

Table 2 shows that more than half of the Norwegians were very satisfied in all issues examined. Less than 30% of the non-western immigrants who described their health as poor were very satisfied with the health services. We know that many of the non-western immigrants are refugees, have complex medical conditions and need specialized services [17]. This group has special needs and might feel that they do not receive the necessary health care.

Younger people were more dissatisfied than older people. The young patients may be more aware of what they can expect from the health services, while on the other hand, older patients may be more reluctant to complain. The people from Sri Lanka were the most satisfied while the Vietnamese were the most dissatisfied. A study in a multicultural area of London showed that Vietnamese patients stated that they had received better health care in general practice than other ethnic groups. But they also stated that they expected less [18]. Reasons for these differences in age and ethnicity are, however, complex, and more research is needed in this field.

We found that 53.7%–63.9% of the non-western immigrants thought they had received the same health services as the Norwegians. These rates are lower than in two previous studies of non-western immigrants in Norway [7,8]. In a 1994 survey of 933 non-western immigrants in Oslo (Tamils, Kurds, Chileans, Iranians, Somalians and Vietnamese), more than 80% of the participants thought they had received the same treatment as the ethnic Norwegians. The percentage was 81.9% in a 1996 survey of 2553 persons from the former Yugoslavia, Turkey, Iran, Pakistan, Vietnam, Sri Lanka, Somalia and Chile. In the last study, 74.5% of the participants thought they had received the medical services they needed.

One explanation of the lower rates in our study, might be differences in data collection methods : The information in the two former studies were based on interviews.

Few of our responders used a translator when visiting a GP. We assume that patients who did not in need a translator either spoke Norwegian or they communicated with a doctor through a mutual language. We also assume that many patients wish to choose a GP who speak their own native language when they do not have a good command of the Norwegian language. In Oslo there are GP's from the non-western parts of the world [19].

In June 2001 the primary health services in Norway were reformed, and the Regular General Practitioner (RGP) Scheme was introduced, based on patient listings and capitation [6]. Parallel to the introduction of the RGP Scheme the number of GPs increased by 300 in Norway. Surveys carried out among Norwegians citizens in 2000 and 2003, showed that the level of satisfaction was high before the reform. The changes were small, but to the better after introduction of the RGP Scheme, probably because of the improved access to GPs [20]. The Oslo Health Study was carried out before introduction of the RGP Scheme, while the Oslo Immigrant Health Study was carried out thereafter. We did, however, not find any major differences between the two groups.

Primary health care services shall treat approximately 90% of the enquiries to health service institutions in Norway. The immigrants' countries of origin have different kinds of health care systems. Thus, the immigrants' different cultural backgrounds may lead to expect admission to a hospital or a hospital's outpatient clinic for conditions that in general are treated by general practitioners in Norway. Other immigrants come from countries without an organised health system. They need information on how to utilise our health care system.

Healthcare experiences as well as health expectations may vary between ethnic groups. In general practice this is especially relevant [18]. Expectations coupled with health needs and experiences are factors that affect patient satisfaction.

Our study has limitations due to small samples. Non-western immigrants are a heterogeneous group, and ethnicity is a complex concept. Belonging to one minority group does not necessarily imply a particular behaviour or a certain health characteristic. Some of the challenges to the health care system include providing services that are sensitive to cultural differences, being able to respond to differences in pattern diseases, and to overcome personal biases [3].

## Conclusion

The non-western immigrants in the two surveys were mostly satisfied with their last visit to a general practitioner. The ethnic Norwegians were overall more satisfied than the non-western immigrants. The rather low response rates opens for the possibility that the degree of satisfaction may not be representative for all immigrants.

This study invites to further research into the non-western immigrants opinions concerning the quality of the primary health care. The aim of further research will be to better understand the non-western immigrants' special needs so as to improve the health services for these patients.

## Competing interests

The author(s) declare that they have no competing interests.

## Authors' contributions

EL and EOR have contributed to the idea, design, analysis and writing of the paper. PN has contributed to analysis and writing of the paper. All authors read and approved the final manuscript.
